# Introducing a novel technique for auricular replantation following subtotal amputation combining Sexton and Mladick pocket methods: a case report

**DOI:** 10.1186/s13256-025-05647-8

**Published:** 2025-11-06

**Authors:** Benedikt Fuchs, Sinan Mert, Constanze Kuhlmann, Nikolaus Thierfelder, Mattis Bertlich, Paul Severin Wiggenhauser

**Affiliations:** 1 Division of Hand, Plastic and Aesthetic Surgery, LMU University Hospital, LMU Munich, Munich, Germany; 2 Department of Dermatology and Allergy, LMU University Hospital, LMU Munich, Munich, 80336 Germany

**Keywords:** Ear amputation, Ear reconstruction, Auricular reconstruction methods, Pocket method, Retroauricular advancement flap, Dog bite, Case report

## Abstract

**Background:**

Traumatic auricular amputations present a unique surgical challenge, particularly in cases where microsurgical anastomosis is not feasible due to vessel damage or absence. Traditional reconstruction techniques often yield suboptimal aesthetic or functional results. The pocket technique, as described by Sexton and Mladick, has shown promise in such scenarios, particularly in partial auricular amputations.

**Case presentation:**

We report a case of a 52-year-old Caucasian male who presented with traumatic amputation of the cranial third of the auricle following a dog bite. The injury was classified as grade III according to the modified Laskin and Donohue grading system. Owing to the absence of suitable vessels for microsurgical repair, a two-stage reconstruction using a novel modification of the pocket technique was performed. In the first stage, under general anesthesia, the skin was carefully dissected from the amputated auricular cartilage, which was then cleaned, debrided, and sutured to the remaining auricle using an “8”-pattern suture technique. A retroauricular advancement flap, adapted from Mladick’s method, was utilized to create a vascularized pocket for the cartilage. After 2 months, the auricle was elevated from the mastoid, and a full-thickness skin graft was applied to cover the defect. A galea advancement flap, based on the posterior branches of the superficial temporal artery, was harvested to provide vascular support and refine the auricular contour.

**Conclusion:**

This case demonstrates the successful application of a modified pocket technique that synergistically integrates principles from Sexton and Mladick’s approaches. The result was a well-perfused, anatomically contoured, and aesthetically pleasing auricular reconstruction without the need for microsurgical anastomosis. This technique represents a viable reconstructive alternative in select cases of partial auricular amputation where vessel quality or availability is compromised.

## Introduction

Auricular trauma occupies a unique position at the intersection of functional, aesthetic, and cultural significance. This was strikingly illustrated by the infamous incident in June 1997, when professional boxer Mike Tyson bit off a portion of Evander Holyfield’s ear during a televised match, drawing worldwide attention to the symbolic and emotional weight carried by the human auricle [1]. Given the broad spectrum of auricular injuries—ranging from minor lacerations to complex avulsion—the need for a structured and nuanced classification system is evident.

To address this variability, we employ a refined classification framework based on the system first introduced by Laskin and Donohue in 1958 [2]. This modified schema stratifies auricular injuries into four grades of severity [3]: grade I involves superficial lacerations with minimal cartilage involvement; grade II encompasses lacerations with a preserved skin bridge; grade III includes avulsions without segment loss, where the detached tissue remains viable for replantation, whether partially or completely separated; and grade IV describes avulsions with segment loss, rendering direct reattachment impossible.

The literature offers a multitude of techniques for auricular reconstruction, each tailored to specific clinical scenarios and resource availability [4–6]. In this context, we present a unique case of partial auricular avulsion successfully treated with an innovative modification of the pocket techniques originally described by Sexton and Mladick *et al*. [7–9]. This report not only contributes to the growing body of reconstructive options for grade III auricular injuries but also underscores the relevance of adapting historical techniques to contemporary clinical practice.

## Case presentation

A 52-year-old Caucasian male presented with traumatic amputation of the cranial third of the auricle due to a dog bite. The amputated segment was appropriately cooled and packed. Given the defect size and lack of suitable vessels for microsurgical anastomosis, we opted for a two-stage reconstruction using a modified pocket technique based on Sexton and Mladick *et al*. [7–9]. This injury, characterized by tearing of a partial segment without complete amputation, is classified as grade 3 in severity according to the modified Laskin and Donohue grading system [2, 3].

**Fig. 1:**
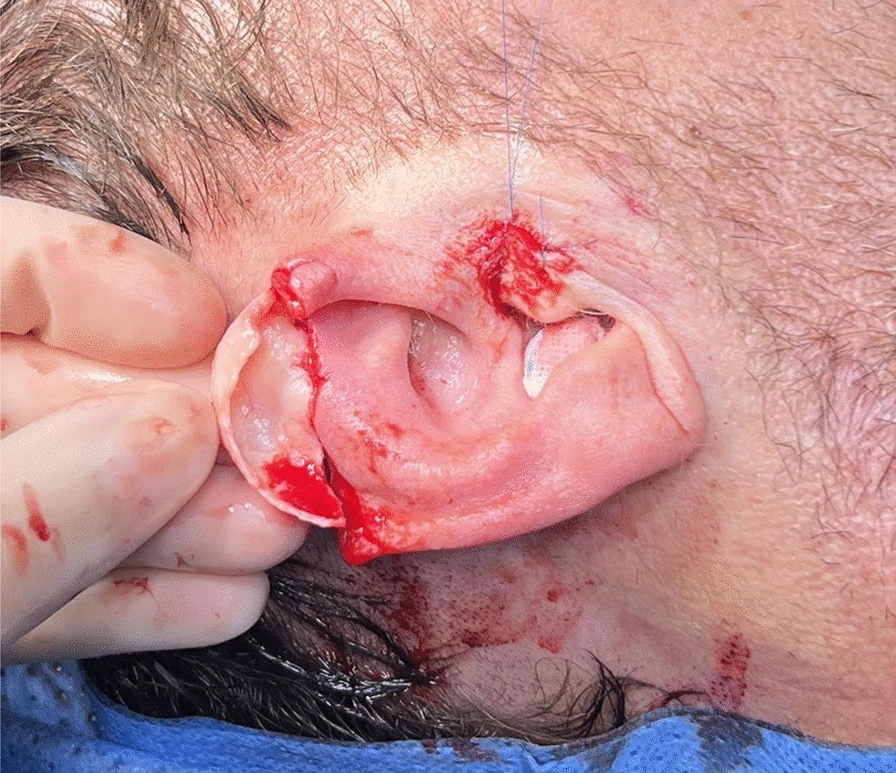
The cleaned auricular cartilage is positioned at the defect site to ensure proper alignment for suturing, enabling complete restoration of the helix’s silhouette

The initial surgical step was performed under general anesthesia, supplemented by local anesthesia via a local ear block. The amputated auricle was repeatedly disinfected. The skin overlying the amputated segment was meticulously dissected from the cartilage using fine scissors under magnification, as described by Sexton [9]. The exposed cartilage was cleansed with Granudacyn^®^, and nonviable tissue was excised. The segment was repositioned and fixed to the remaining auricle using 4–0 and 5–0 PDS II^®^ sutures in an “8” pattern, ensuring optimal integration and anatomical contouring (Fig. 1).

**Fig. 2:**
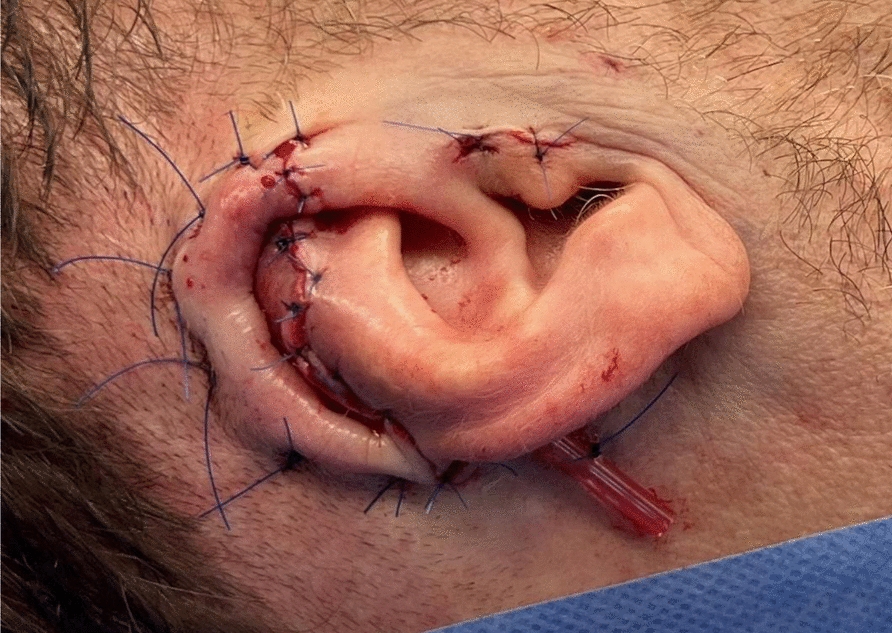
Preparation of the subcutaneous pocket over the mastoid region and placement of the cartilage replant. Placement of the replant using mattress sutures to contour the scapha and ensure adequate contact between the overlying skin and the cartilage tissue. In addition, an Easy-Flow^®^ drain was inserted to facilitate continuous drainage and prevent hematoma formation

A retroauricular advancement flap was then designed and elevated from the mastoid bone. The flap was advanced to cover the cartilage and sutured using 5–0 Prolene^®^ for the skin and horizontal mattress sutures (3–0 Prolene^®^) to secure the skin to the cartilage (Fig. 2). This adaptation, derived from Mladick’s pocket technique, improved cartilage nutrition and epithelialization. A small drain was inserted to prevent hematoma formation and removed on postoperative day two. A compression dressing ensured proper molding of the scapha and antihelix.

**Fig. 3:**
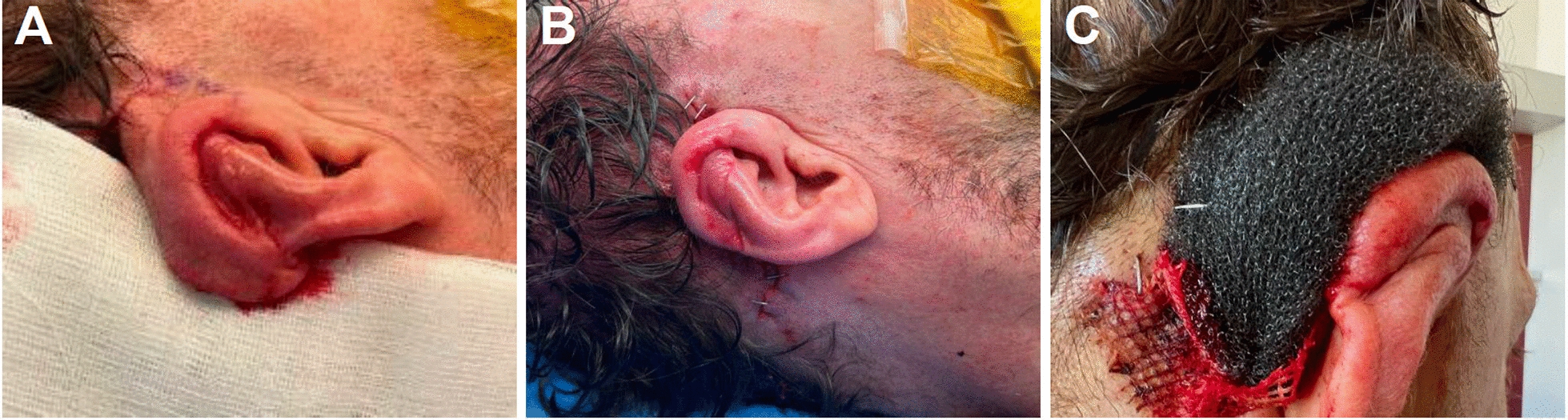
**A** An advancement galea flap from the temporoparietal fascia was harvested to cover the posterior auricular surface, with a cranially marked Burrow’s triangle, ensuring adequate perfusion and a healthy rosy appearance throughout the procedure. **B** The flap was folded posteriorly to cover the back of the ear and sutured in place without tension. Wound closure on the lateral and medial aspects was achieved using stapled sutures. The donor site was covered with a full-thickness skin graft. **C** To ensure adequate perfusion and nutrient diffusion of the full-thickness skin graft during the critical first 72 hours postoperatively, a tie-over dressing with mild compression was applied

At 2 months postoperatively, the replanted segment exhibited successful integration. Under general anesthesia, the auricle was surgically elevated from the mastoid, and the resultant defect was reconstructed using a full-thickness skin graft (5 × 4 cm) harvested from the right upper arm. To refine auricular contour and ensure vascular support, a galea flap was designed to encircle the helix. The flap, tapering cranially to caudally, was perfused by posterior branches of the superficial temporal artery. The incision was made, and the galea flap was elevated from the temporoparietal fascia (Fig. 3). The cranial helix remained well-perfused. The auricle was lifted from the mastoid base, and an additional advancement flap was mobilized by undermining the galea above the temporoparietal fascia. Small incisions in the temporoparietal fascia facilitated better mobilization, and the flap was secured to the posterior helix using 2–0 and 4–0 Monocryl^®^ sutures. Cranial anchoring of the auricle to the descending mastoid ensured stable positioning. A Burrow’s triangle was incorporated at the cranial helix base to optimize contouring. The advancement flap was placed cranially and sutured using 2–0 and 4–0 Vicryl^®^ in the deep layers. The remaining retroauricular defect (5 × 4 cm) was covered using the full-thickness skin graft, which was fenestrated and secured with interrupted 4–0 Monocryl^®^ sutures. A compression dressing with ointment-soaked gauze and a vacuum sponge was applied for optimal graft adherence.

**Fig. 4:**
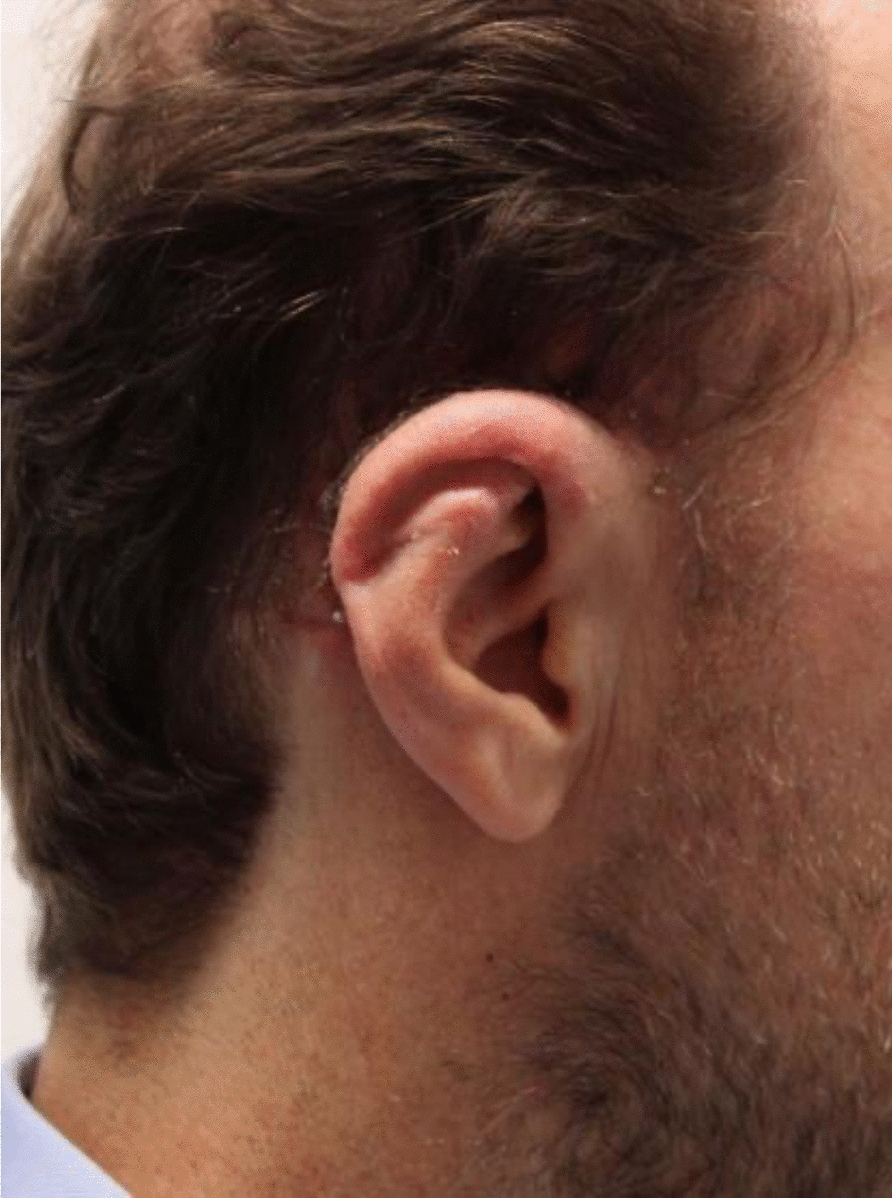
Postoperative outcome after 3 weeks. The partial cartilage segment demonstrates successful healing, with the overlying skin appearing vital, rosy, and well-perfused. The full-thickness skin graft covering the lifting defect has completely healed. The helix, crus superior of the anthelix, and scaphoid fossa are well-defined. The angle between the mastoid plane and auricular plane is 25°, and the distance from the mastoid plane to the upper edge of the helix is 18 mm

Postoperatively, the reconstructed auricle exhibited good perfusion, preserved anatomical contour, and a satisfactory aesthetic outcome (Fig. 4).

## Discussion

To address the variability and complexity of auricular injuries, we use a modified classification system based on the framework established by Laskin and Donohue in 1958 [2, 3]. Particular emphasis is placed on grade III injuries (complete amputation without segment loss), as timely intervention can enable replantation of the detached auricular tissue, yielding promising functional and aesthetic outcomes. The literature contains numerous different methods for reconstructing the auricle. As reported in previous studies, ear reconstruction using the pocket method yields optimal outcomes in cases of partial, rather than complete, amputation when no suitable vessels are available for microsurgical anastomosis [10].

This case report introduces a novel modification of the ear reconstruction technique based on the pocket methods described by Sexton *et al*. and Mladick *et al*. [7, 9]. This new approach integrates both reconstruction techniques to treat a single patient with traumatic auricular amputation. We advocate for the complete separation of the skin and subcutaneous tissue from the auricular cartilage, as outlined by Sexton, to enhance cartilage nutrition and ensure optimal adaptation of the amputated cartilage to the residual auricular framework. Simultaneously, Mladick’s modified approach, which incorporates mattress sutures, proves advantageous in restoring the aesthetic anatomical units and contours of the ear while further improving cartilage viability. The modified pocket technique, which synergistically combines the methodologies of Sexton *et al*. and Mladick *et al*., provided a practical and aesthetically favorable solution for auricular reconstruction in this patient.

## Conclusion

This case illustrates a novel approach to auricular reconstruction by integrating two established pocket techniques—those of Sexton and Mladick—into a unified method for managing a grade III auricular avulsion. The successful anatomical and aesthetic outcome highlights the potential of this modified technique as a viable alternative in cases where microsurgical replantation is not feasible.

## Data Availability

Not applicable.
